# No system-size anomalies in entropy of bcc iron at Earth’s inner-core conditions

**DOI:** 10.1038/s41598-018-25419-3

**Published:** 2018-05-08

**Authors:** Andrew J. Schultz, Sabry G. Moustafa, David A. Kofke

**Affiliations:** Department of Chemical and Biological Engineering, University at Buffalo, The State University of New York, Buffalo, New York 14260-4200 USA

## Abstract

New molecular modeling data show that the entropy of bcc iron exhibits no system-size anomalies, implying that it should be feasible to compute accurate free energies of this system using first-principles methods without requiring a prohibitively large number of atoms. Conclusions are based on rigorous calculations of size-dependent free energies for a Sutton-Chen model of iron previously fit to ab initio calculations, and refute statements recently appearing in the literature indicating that the size of the simulation cell is critical for stabilization of the bcc phase.

## Introduction

Recently, data from molecular simulations of iron at Earth’s inner-core conditions have been reported in ref.^1^ (hereafter referred to as (I)). A key focus of (I) is the stability of the bcc phase relative to hcp at these conditions. A major claim made there is that a diffusion mechanism stabilizes bcc, and that simulations of very large systems are needed for this effect to be realized. Several observations are made in (I) to provide evidence for this conclusion. Primarily it is found that isobaric molecular dynamics (MD) simulations of atomistic models of iron using density functional theory (DFT) spontaneously transition from bcc to hcp when simulated using a small number of atoms (*N* = 432), but for larger systems (*N* = 1024) such transitions are not observed for the duration of 18-ps simulations at 7000 K. However, at lower temperature (6000 K), the 1024-atom simulations also spontaneously transition to hcp, and it is argued that it is not possible to determine whether this is still a system-size effect, or the true behavior for this temperature. *Ab initio* simulations of larger systems needed to address this are not presently feasible. Examination of configurations and trajectories in (I) shows concerted diffusive processes, and the authors of (I) assert that this behavior contributes to the entropy and thereby stabilizes bcc; moreover they state that such processes cannot be supported by small systems without destroying their structure. This then, according to (I), is the origin of the strong size dependence of the bcc stability.

To examine this phenomenon further, the authors of (I) parameterized a Sutton-Chen (SC) embedded-atom model by fitting to DFT energies for select configurations of bcc and hcp phases observed in 1024-atom simulations at 6000 K. Energies and forces from SC are much less computationally expensive to evaluate compared to DFT, so the SC system could be simulated for much larger system sizes; results from simulations of up to 16 × 10^6^ atoms were reported in (I). The same behavior is observed in the SC calculations as seen for DFT: the system spontaneously transitions from bcc to hcp when simulating smaller systems and lower temperatures, while this transition is not observed over the duration of simulations of systems with greater numbers of atoms. The bcc phase is observed to persist for at least 200 ps in 65,536-atom simulations for temperatures as low as *T* = 5500 K (but not 5000 K). We have independently performed a more limited set of simulations of the SC model and have observed the same general behaviors as described in (I).

**Figure 1:**
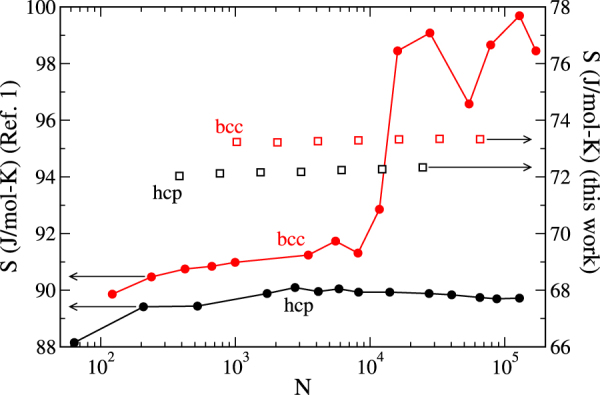
Entropy for bcc (red) and hcp (black) phases of iron as a function of simulated-system size at 360 GPa and 7000 K. Filled symbols joined by lines (left axis) are results from (I); open symbols (right axis) are results computed here, with estimated stochastic + systematic errors that are smaller than symbol size. Note that left and right axes are shifted with respect to each other, but cover the same range (12 J/mol-K).

To put the observations on a thermodynamic footing, the authors of (I) collected averages of the SC hcp and bcc dynamics and used these data in an approximate method, based on analysis of the velocity autocorrelation function (VACF), to estimate the entropy of each phase. Their results for the bcc entropy per mole *S*_b*cc*_/*N* show a remarkable dependence on system size (Fig. 1), which the authors of (I) use to explain the increase in mechanical stability of bcc with *N*. The strong variation of the entropy with system size (even up to *N* = 20,000) violates the expected linear scaling that is characteristic of an extensive thermodynamic quantity (in contrast, the hcp entropy per mole varies little with *N*). This unusual behavior is ascribed to the enhanced bcc diffusion observed in both the DFT- and the SC-based studies, which is manifested only for very large *N*. The implication is that simulations of small systems are unable to capture the essential phenomena that govern the thermodynamic stability of bcc iron.

The issue of a non-extensive entropy for moderate *N* is important, because a need for very large *N* to capture bcc behavior correctly would preclude evaluation of the entropy using an accurate DFT model, which is too computationally expensive for the large system sizes prescribed by (I). If true, this means that we may give up any hope of computing an accurate phase diagram for iron at extreme conditions from first-principles considerations.

Of course, diffusion *per se* does not contribute to the free energy, which is an equilibrium thermodynamic property that can be defined and computed without any reference to dynamic behavior. Hence, it should be possible to demonstrate the anomalous entropy scaling just as well through rigorous free-energy calculations using any convenient simulation method. For force-field models (such as SC) it is possible to evaluate the free energy very accurately (within any approximation inherent in the molecular model), enabling the observations made in (I) to be rigorously tested. It is still a challenging calculation however, because at the pressures of interest bcc is mechanically (and thus thermodynamically) unstable at *T* = 0 K, so we cannot apply a conventional approach based on integration from low temperature^2^; further, results from methods using integration from a noninteracting cell model or Einstein lattice^3^ should work but might be viewed with some doubt because they completely suppress diffusion along part of the integration path, and the switch from non-diffusive to diffusive behavior could be problematic.

Nevertheless, we have completed such calculations, generating very accurate entropies for the SC model developed in (I), for different system sizes *N*. The approach we take to calculate the *N*-dependent Helmholtz free energy is to perform successive calculations in which we compute the difference Δ*A*(*N*) in free energy upon doubling of the system size, Δ*A*(*N*) ≡ *A*(*T*, 2*V*, 2*N*) − *A*(*T*, *V*, *N*). This type of calculation has been proposed previously^4–6^ as a means to obtain the absolute free energy, by coupling the result with the assumption that the free energy is a first-order homogeneous function of *V* and *N*: *A*(*T*, 2*V*, 2*N*) = 2*A*(*T*, *V*, *N*) (i.e., the free energy is an extensive thermodynamic quantity). In the present case, our primary interest is to test whether the free energy is extensive for small-to-moderate values of *N*, so instead we perform this calculation for successively larger systems, repeatedly doubling the system size, and we examine whether the successive differences are consistent with an extensive free energy. The treatment is appealing because the calculated free energy differences coincide with the independent variable of interest (*N*), and moreover the system is at all stages stable (or at least metastable) and free to undergo some diffusive processes.

We find that the free energy is indeed extensive, and there is no anomalous behavior of *S*_b*cc*_ with system size. Results are presented in the next section, followed by a discussion, and a description of the methods used.

## Results

Our calculations yield absolute free-energy values including all contributions (i.e. lattice, harmonic, and anharmonic) due to motion of the nuclei according to the Sutton-Chen semi-empirical potential given in (I). Inasmuch as the SC potential was fit to DFT calculations using Fermi-Dirac occupation (smearing), results obtained from the model in principle include electronic excitation contributions, hence they do not need to be accounted for separately.

We performed system-doubling simulations to compute free energy differences Δ*A*(*N*_*j*_) for *N*_*j*_ → 2*N*_*j*_, with *N*_*j*_ as small as 192 atoms and as large as 32,768 (doubling to 65,536), as detailed in the methods section. The Δ*A*(*N*_*j*_) values for bcc and hcp phases were computed from equation (16). Now, if Δ*A*(*N*_*j*_)/*N*_*j*_ is independent of *N*_*j*_, then growth of *A* with *N* (as given by successive increments in Δ*A*) is linear in *N*, and hence *A* is extensive. Therefore, to gauge whether *A* is extensive, we may examine Δ*A*(*N*_*j*_)/*N*_*j*_ to see whether it is constant with *j*. Apart from this, we note also that for an extensive free energy, *A*(*N*_*j*_)/*N*_*j*_ = Δ*A*(*N*_*j*_)/*N*_*j*_, so as we examine the variation in Δ*A*(*N*_*j*−1_)/*N*_*j*−1_, it is useful to regard it in terms of *A*(*N*_*j*_)/*N*_*j*_ itself, thereby allowing comparison to the free energy values reported in (I). Thus, in Table 1 we report the values of Δ*A*(*N*_*j*−1_)/*N*_*j*−1_ obtained as described above, but we label them in the table as *A*(*N*_*j*_)/*N*_*j*_.

**Table 1:** Thermodynamic properties computed in this work.

*N*	bcc				hcp			
	*A*/*Nk*_B_*T*	〈*U*〉/*Nk*_B_*T*	*S*/*Nk*_B_	*G*/*Nk*_B_*T*	*A*/*Nk*_B_*T*	〈*U*〉/*Nk*_B_*T*	*S*/*Nk*_B_	*G*/*Nk*_B_*T*
*N*_1_	−17.4380(12)	−8.636(6)	8.802(6)	7.3330(12)	−17.3387(18)	−8.673(8)	8.665(8)	7.3051(18)
*N*_2_	−17.4323(13)	−8.623(3)	8.809(3)	7.3386(13)	−17.3435(18)	−8.688(6)	8.656(6)	7.3003(18)
*N*_3_	−17.445(2)	−8.6214(18)	8.823(3)	7.326(2)	−17.344(2)	−8.684(3)	8.660(4)	7.299(2)
*N*_4_	−17.448(5)	−8.616(2)	8.831(5)	7.323(5)	−17.349(2)	−8.685(3)	8.664(4)	7.295(2)
*N*_5_	−17.450(6)	−8.6223(12)	8.827(7)	7.321(6)	−17.348(3)	−8.6775(18)	8.670(3)	7.296(3)
*N*_6_	−17.443(9)	−8.6152(10)	8.827(9)	7.328(9)	−17.347(4)	−8.6838(16)	8.663(4)	7.297(4)
*N*_7_	−17.453(11)	−8.6189(8)	8.834(11)	7.318(11)	−17.346(4)	−8.6793(12)	8.666(4)	7.298(4)

*N*_*j*_ is the system size as defined in Table 3; *A* is the Helmholtz free energy; 〈*U*〉 is the internal potential energy averaged in the *w* = 0 limit; *S* is the entropy; *G* is the Gibbs free energy, assuming a pressure of 360 GPa. Numerals in parentheses indicate the estimated error (stochastic + systematic; see text) in the rightmost digit(s) of the reported value. All data are for *T* = 7000 K and specific volumes of 6.65 Å^3^/atom for the bcc phase, and 6.616 Å^3^/atom for hcp.

We compute the entropy *S* from the Helmholtz free energy via

1 $$S=\frac{\langle U\rangle -A}{T},$$

which gives us the quantity plotted as the open points in Fig. 1. Here, the average energy is determined from the simulations (with *w* = 0; see Methods section). This average and all other thermodynamic properties computed here are recorded in Table 1 for each system size and crystal structure.

The Gibbs free energy indicates whether hcp or bcc is more stable, with both at 7000 K and 360 GPa. This is obtained via *G*(*N*) = *A*(*N*) + *PV*, and is computed for each phase as a function of system size and included in Table 1.

## Discussion

Considering Fig. 1, our findings agree with (I) that the entropy favors bcc relative to hcp, but the difference is much smaller than reported for the *N* > 20,000 systems in (I), which is taken by the authors of (I) to be where the true bcc behavior is manifested. The discrepancy almost certainly stems from their use of the VACF to estimate the entropy, an approach that must fail when diffusive behavior becomes significant. The weakness of using VACF is acknowledged in (I), which is careful to indicate that the large *S*_b*cc*_ represents an upper bound of possible values, while concluding it nonetheless reflects essential behavior that necessitates large-*N* simulations to compute *S*_b*cc*_ rigorously. This conclusion is refuted by our results, which finds little or no variation in *S*/*N* with *N* for the bcc (and hcp) phase, for *N* as small as 512 and as large as 32,000 atoms.

In addition to the incompatibility of our entropy results and the VACF calculations with respect to variation with *N*, the absolute magnitudes of the entropies from the two methods differ considerably: the VACF values are larger than the entropies that we compute by about 20 J/mol-K. Notably however, the intermediate-*N* VACF estimate of the bcc-hcp entropy *difference* is in very good agreement with the difference computed from our data. Of course, there is a large region of system sizes where the VACF estimate of the difference is inaccurate, so one must take care not to treat the partial agreement with our results as suggesting that VACF methods are generally reliable in providing entropy differences.

Table 2 records differences in key thermodynamic properties between the phases; these are evaluated directly from the data in Table 1. Enthalpy favors hcp (due mainly to the *PV* contribution), and the entropy difference we compute is not quite sufficient to overcome this to yield a bcc-favorable Gibbs free energy; within uncertainty, they could be in equilibrium at 7000 K and 360 GPa. Undoubtedly then for temperatures *T* < 7000 K the bcc phase for this SC iron model is less stable than hcp, because the enthalpy advantage of hcp becomes even more dominant at lower temperatures. We speculate that evidence in (I) for growth of bcc stability with *N* based on its persistence in large-system simulations is due to growth of the bcc → hcp activation barrier with *N*, and its reduction with *T*; however, more detailed calculations would be needed to prove this explanation. In any case, rigorous free-energy calculations as an indicator of relative stability must be considered more definitive than conclusions based on the absence of spontaneous transformation.

**Table 2:** Differences *δ* ≡ (bcc) − (hcp) in thermodynamic properties for the Sutton-Chen model defined in (I).

Source	*δA*/*Nk*_B_*T*	*δ* 〈*U*〉/*Nk*_B_*T*	*PδV*/*Nk*_B_*T*	*δH*/*Nk*_B_*T*	*δS*/*Nk*_B_	*δG*/*Nk*_B_*T*
This work	−0.107(15)	0.0604(14)	0.1272	0.1876(14)	0.167(15)	0.020(15)
Ref.^1^					(0.345, 0.962)	

“This work” indicates results as computed in this work, using property values for the systems of size *N*_6_ for each phase. Entropy is reported in ref.^1^ as a range of values, represented here by the pair of numbers in parentheses. *A* is the Helmholtz free energy; 〈*U*〉 is the energy; *H* is the enthalpy; *S* is the entropy; *G* is the Gibbs free energy, *P* = 360 GPa is the pressure, *V*/*N* is the volume per atom (6.65 Å^3^/atom for the bcc phase, and 6.616 Å^3^/atom for hcp). Numerals in parentheses indicate the estimated error (68% confidence) in the rightmost digit(s) of the reported value.

Our results regarding bcc/hcp stability for the SC model, while rigorous, cannot be considered conclusive with respect to real iron at 7000 K. First, the SC model was fit to DFT energies generated at only one temperature (6000 K), while our analysis is performed at 7000 K to allow direct comparison with SC data reported in (I). Apart from the inaccuracy introduced by not including in the fit the effect of differences in sampling at different states, the effect of temperature on the electronic excitation contribution of the potential is not characterized by the fit as well. In addition, even at 6000 K the SC model exhibits significant differences from accurate *ab initio* DFT calculations for the energy and pressure of crystalline iron, despite being fit to DFT calculations at this temperature. Specifically, isothermal-isobaric simulations we conducted using LAMMPS at 6000 K and 360 GPa with the SC model yielded an average energy (〈*U*〉 = −5360 eV (bcc) and −5490 eV (hcp) for 1024 atoms) that differs from the range of energies used in (I) to fit the potential (roughly −5550 to −5750 eV for 1024 atoms, across both bcc and hcp phases). The configurations used in (I) to perform the fit were generated by sampling the DFT potential. Hence, despite its accurate reproduction of the energies of the fitted configurations, this difference in 〈*U*〉 suggests that samples generated by the SC model differ from those obtained from the DFT potential it is fit to, even when both are sampled at the same temperature.

Finally, we make a few points related to diffusion in this system. We observed in our simulations the cooperative diffusion events that were reported in (I); these occurred in some systems more than others. A cursory study of the trajectories suggests that they represent the spontaneous generation of a vacancy-interstitial pair that move away from each other after creation. They eventually recombine to stop the process. This recombination occurs more quickly in small systems than in larger ones, which might explain why the phenomenon is observed more in the larger systems. Regardless of the nature of the diffusion events, we find no obvious correlation between the amount of diffusion and the results we obtain for the thermodynamic properties. In fact, we conducted a full study very similar to the one described here, but with a much stronger thermostat (and, incidentally, using a different form for *ϕ* (Eq. (8a)). The stronger thermostat had the (unintended) effect of completely suppressing diffusion. Concerned that this might be perceived as undermining relevance to (I), we repeated all calculations with the weaker thermostat described here, which allows for significant diffusion. The results of the two studies are identical within statistical uncertainty–the presence or absence of diffusion has no effect on the entropy.

In summary, the VACF approach used in (I) fails for systems with diffusion (which is more prevalent for larger *N*) and yielded inaccurate free energies, contributing to the idea that prohibitively large systems are needed to simulate bcc iron properly. Instead, our findings indicate that the bcc entropy per mole is, as expected, nearly constant with system size down to 512 atoms. Consequently it it should be possible to compute accurate free energies of bcc iron without requiring prohibitively large system sizes.

## Methods

### Free-energy calculations

The free energy difference Δ*A*(*N*) for a given *N* → 2*N* case is computed via thermodynamic integration. The system is at all points in the integration path composed of 2*N* atoms in a volume of size 2*V*. The simulation volume is partitioned conceptually into two equal rectangular subvolumes, each of size *V*; for specificity, we state that one subvolume is translated a distance *L*_*x*_ in the *x* coordinate direction with respect to the other subvolume, where *L*_*x*_ is the *x*-edge length of each subvolume. Each atom in one subvolume is coupled to a partner atom in the other; label the mutual partners *i* and *i* + *N*. Nominally, the partner atoms are at positions where they would be periodic images of each other, and the degree to which this relation is imposed is characterized by the integration parameter *λ*. At the start of the integration path, *λ* = ∞ and the partner atoms are rigidly coupled, so the configurations sampled within the two subvolumes are identical, and one is truly a periodic image of the other. At the end of the integration path, *λ* = 0 and the partner atoms are completely uncoupled. We will use *A*_*λ*_ to indicate the *λ*-dependent free energy along the path.

The system obtained at the “no-constraint” end of the path is a conventional one having free energy *A*(*T*, 2*V*, 2*N*); thus

2 $${A}_{0}(T,\,2V,\,2N)=A(T,\,2V,\,2N)$$

At the other “rigid” end of the path, the system is closely related to one with *N* particles in a volume *V*, except that the energy of each configuration is exactly doubled, because it has identical contributions from both subvolumes. To offset this, we double the temperature as well, thus:

3 $${A}_{\infty }\mathrm{(2}T,\,2V,\,2N)=A(T,\,V,\,N)$$

Consequently, we require variation of temperature along the path, following *τ*(*λ*), such that *τ*(0) = *T* and *τ*(∞) = 2*T*. In terms of the partition function, the free energies are

4a $$\exp (\,-\,{A}_{0}/{k}_{{\rm{B}}}T)=\frac{1}{\mathrm{(2}N)!{\rm{\Lambda }}{(T)}^{6N}}Z(\tau \mathrm{(0),}\,2V,\,2N;\varphi \mathrm{(0)})$$

4b $$\exp (\,-\,{A}_{\infty }/{k}_{{\rm{B}}}T)=\frac{1}{{2}^{N}N!{\rm{\Lambda }}{(T)}^{3N}}Z(\tau (\infty ),\,2V,\,2N;\varphi (\infty )),$$

where Λ(*T*) is the de Broglie thermal wavelength, and the *λ*-dependent configurational integral is defined:

5 $$Z(\tau (\lambda ),\,2V,\,2N;\varphi (\lambda ))={\int }_{2V}\exp [-\frac{1}{{k}_{{\rm{B}}}\tau (\lambda )}(U({{\rm{r}}}^{2N})+\sum _{i\mathrm{=1}}^{N}\varphi ({{\rm{r}}}_{i},{{\rm{r}}}_{i+N};\lambda ))]d{{\rm{r}}}^{2N};$$

the factor of 2^*N*^ appearing in equation (4b) accounts for the indistinguishability of the partner atoms. Here we have introduced the pair function *ϕ*(r_*i*_, r_*i* + *N*_; *λ*), which imposes the partner coupling. Consistent with the integration limits, *ϕ*(r_*i*_, r_*i* + *N*_; 0) ≡ 0, and exp[−*ϕ*(r_*i*_, r_*i* + *N*_,∞)] = *δ*(**r**_*i* + *N*_ − **r**_*i*_ − **L**), where *δ* is the Dirac delta function and **L** is the vector (*L*_*x*_, 0, 0). Conventional periodic boundary conditions are applied to the (2*N*, 2*V*) system for all *λ*, so all energy functions (including *ϕ*) are evaluated using the minimum-image convention.

Combining the above, the working equation for the desired free energy difference is

6 $$\frac{{\rm{\Delta }}A(N)}{{k}_{{\rm{B}}}T}=\,\mathrm{ln}\,\frac{\mathrm{(2}N)!}{{2}^{N}N!}+3N\,\mathrm{ln}\,{\rm{\Lambda }}(T)+{\int }_{0}^{\infty }\frac{d\,\mathrm{ln}\,Z}{d{\lambda }}d{\lambda }.$$

The integration is performed to a value of *λ* that is large enough to ensure that the partner-atom separation is nearly rigid, meaning that *U* does not vary significantly for any observed deviations from rigidity. Designating this value *λ*_m_, equation (6) becomes

7 $$\frac{{\rm{\Delta }}A(N)}{{k}_{{\rm{B}}}T}=\,\mathrm{ln}\,\frac{\mathrm{(2}N)!}{{2}^{N}N!}+3N\,\mathrm{ln}\,{\rm{\Lambda }}(T)+{\int }_{0}^{{{\lambda }}_{{\rm{m}}}}\frac{d\,\mathrm{ln}\,Z}{d{\lambda }}d{\lambda }-N\,\mathrm{ln}\,\int {e}^{-\varphi \mathrm{(0,}r;{{\lambda }}_{{\rm{m}}})/{k}_{{\rm{B}}}\tau ({{\lambda }}_{{\rm{m}}})}d{\bf{r}};$$

the **r** integral appearing here is the ratio *Z*(*τ*(*λ*_m_), 2*V*, 2*N*; *λ*_m_)/*Z*(2*T*, 2*V*, 2*N*, ∞) (which assumes *τ*(*λ*_m_) ≈ 2*T*); the integrand diminishes rapidly with |**r**|, so this integral may be taken over an infinite volume.

A natural choice for the constraint function *ϕ* is a harmonic spring, with width given in terms of *λ*:

8a $$\varphi ({{\rm{r}}}_{1},{{\rm{r}}}_{2};{\lambda })=\varepsilon w({\lambda }){r}^{2}$$

8b $${r}^{2}={(|{r}_{x\mathrm{,1}}-{r}_{x\mathrm{,2}}|-{L}_{x})}^{2}+{({r}_{y\mathrm{,1}}-{r}_{y\mathrm{,2}})}^{2}+{({r}_{z\mathrm{,1}}-{r}_{z\mathrm{,2}})}^{2}$$

where we take *ε* = 10 J/mol. The rigidity parameter *w* appearing in equation (8a) is selected as a function of *λ* to support the performance of the free energy calculation. After some trial-and-error, we chose the form satisfying:

9 $${\lambda }=\,\mathrm{ln}(1+bw),$$

where *b* = *e*^−4^ Å^2^ is chosen to enhance the smoothness of *d*ln*Z*/*dλ*; equation (9) may be easily inverted to obtain *w*(*λ*).

For *ϕ* given by equation (8a), the r integral in equation (7) is

10 $$\int {e}^{-\varphi (0,{\rm{r}};{\lambda }_{{\rm{m}}})/{k}_{{\rm{B}}}\tau ({\lambda }_{{\rm{m}}})}d{\bf{r}}={(\frac{\pi {k}_{B}\tau ({\lambda }_{{\rm{m}}})}{\varepsilon w({\lambda }_{{\rm{m}}})})}^{3/2}.$$

The temperature protocol *τ*(*λ*) is constructed using the heuristic of keeping the average energy 〈*U*〉_*λ*_ roughly constant along the path. This is done also by trial-and-error. We use the following form:

11 $$\tau ({\lambda })=T(1+\frac{w({\lambda })}{{[{a}_{0}+{a}_{1}w({\lambda })+w{({\lambda })}^{2}]}^{\mathrm{1/2}}})$$

where *a*_0_ = 1.2253 × 10^8^ Å^−4^ and *a*_1_ = 416305 Å^−2^. The same protocol *τ*(*λ*) was used for all system-size doublings for both bcc and hcp.

Finally, the integrand for the thermodynamic integration in *λ* is given via the ensemble averages (indicated by angle brackets):

12 $$\begin{array}{rcl}f({\lambda };N) & \equiv  & \frac{d\,\mathrm{ln}\,Z}{d{\lambda }}-\frac{\bar{U}}{{k}_{{\rm{B}}}{\tau }^{2}}(\frac{d\tau }{d{\lambda }})\\  & = & ({\langle \frac{U-\bar{U}}{{k}_{{\rm{B}}}{\tau }^{2}}\rangle }_{{\lambda }}+{\langle \frac{{\rm{\Phi }}}{{k}_{{\rm{B}}}{\tau }^{2}}\rangle }_{{\lambda }})(\frac{d\tau }{d{\lambda }})+{\langle -\frac{1}{{k}_{{\rm{B}}}\tau }\frac{d{\rm{\Phi }}}{d{\lambda }}\rangle }_{{\lambda }}\end{array}$$

where Φ is the total constraining potential:

$${\rm{\Phi }}\equiv \sum _{i\mathrm{=1}}^{N}\varphi ({{\bf{r}}}_{i},{{\bf{r}}}_{i+N};\lambda ),$$

and $\bar{U}$ is the mean of the energy-averages across the path:

13 $$\bar{U}\equiv \frac{1}{{n}_{{\lambda }}}\sum _{i\mathrm{=1}}^{{n}_{{\lambda }}}{\langle U\rangle }_{{{\lambda }}_{i}}.$$

We subtract $\bar{U}$ when defining *f* because the *τ*(*λ*) protocol is designed to keep 〈*U*〉_*λ*_ roughly constant across the integration path. Thus, 〈*U*−$\bar{U}$〉_*λ*_ is nearly zero for all *λ* and the first of the three terms on the right-hand side of equation (12) is small. This is good because (*dτ*/*dλ*) is not small and has significant curvature, and consequently it would introduce inaccuracy in the quadrature. The bulk of the contribution from integrating (*U*/*k*_B_*τ*^2^) (*dτ*/*dλ*) is captured by ($\bar{U}$/*k*_B_*τ*^2^) (*dτ*/*dλ*), for which the integral can be evaluated analytically:

14 $${\int }_{0}^{\infty }\frac{\bar{U}}{{k}_{{\rm{B}}}{\tau }^{2}}\frac{d\tau }{d{\lambda }}d{\lambda }=\frac{1}{2}\frac{\bar{U}}{{k}_{{\rm{B}}}T}.$$

We define ${\mathscr{J}}(N,{\lambda }_{{\rm{m}}})$ as the integral of *f*:

15 $${\mathscr{J}}(N,{{\lambda }}_{{\rm{m}}})\equiv {\int }_{0}^{{{\lambda }}_{{\rm{m}}}}f({\lambda };N)d{\lambda }.$$

Then in terms of this the free energy difference is:

16 $$\frac{{\rm{\Delta }}A(N)}{{k}_{{\rm{B}}}T}=\,{\rm{l}}{\rm{n}}\,\frac{(2N)!}{{2}^{N}N!}+\frac{1}{2}\frac{\bar{U}}{{k}_{{\rm{B}}}T}+{\mathscr{J}}(N,{\lambda }_{{\rm{m}}})+\frac{3}{2}N\,{\rm{l}}{\rm{n}}(\frac{\varepsilon w({\lambda }_{{\rm{m}}}){{\rm{\Lambda }}}^{2}(T)}{\pi {k}_{B}\tau ({\lambda }_{{\rm{m}}})})$$

**Figure 2:**
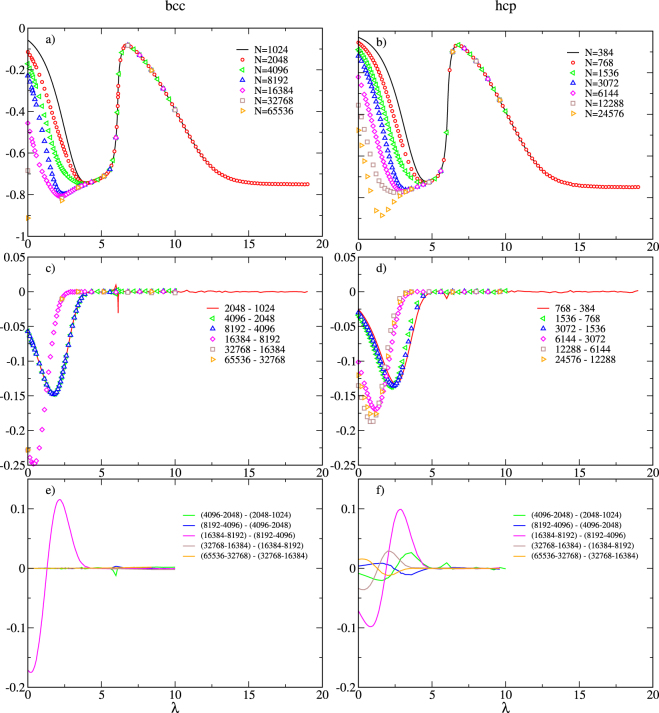
Integrand of equation (12), *f*(*λ*, *N*) (top row) and its first and second differences *f* ^(1)^(*λ*, *N*) (middle row), and *f* ^(2)^(*λ*, *N*) (bottom row), respectively. Note that, according to Eq. (20), only the smallest-system *f*(*λ*, *N*) data are integrated (black line in top-row plots), and *f* ^(1)^(*λ*, *N*) data are integrated for only the smallest pair of systems (red line in middle-row plots). All *f* ^(2)^(*λ*, *N*) are integrated. Lines in each figure join points not represented by symbols (no symbols are joined by lines).

The integrand *f*(*λ*; *N*) given by equation (12) as computed by molecular simulation is plotted in Fig. 2, parts (a) and (b) for the bcc and hcp systems, respectively. At large *w* there is no observable difference with system size, but for smaller *w* the integrands for a given allotrope differ noticably with *N*. This variation is expected because for larger boxes the atoms have more space to sample, and this factors into the average for smaller *w*, where the finite box volume rather than *ϕ* limits partner separations. The *N* variation in the integral is compensated by the combinatorial terms involving *N*! in equation (6), and the overall free energy difference becomes essentially *N*-independent.

The integrands *f*(*λ*; *N*) exhibit significant curvature, which introduces inaccuracy in the integrals when quadrature is performed. Given that we are interested in differences in the integrals with respect to *N*, we can offset the problems with curvature by working with differences in the integrands. We find that it is useful to consider up to second differences. Thus, in parts (c) and (d) of Fig. 2 we plot the differences

17 $${f}^{\mathrm{(1)}}({\lambda };N)\equiv f({\lambda };\,2N)-f({\lambda };N),$$

while in parts (e) and (f) we present

18 $${f}^{\mathrm{(2)}}({\lambda };N)\equiv {f}^{\mathrm{(1)}}({\lambda };\,2N)-{f}^{\mathrm{(1)}}({\lambda };N\mathrm{)}.$$

The plots show that these first and second differences are significantly smaller in magnitude than *f*(*λ*; *N*) itself and have a much narrower range of *λ* where they are nonzero. The second difference *f*^(2)^ shows a significant dependence on system size, with some noticeable variation in magnitude and shape for the different *N*. This variation is a result of the variation in the aspect ratio of the box (cubic/oblate/prolate) through successive system doublings. The same behavior was observed in our tests of the method on the fcc Lennard-Jones model, so it does not signify any unusual features of iron or the crystal structures being studied here. The differences average out upon integration over *λ*.

We can define a corresponding set of differences in the integrals:

19a $$\begin{array}{ccc}{{\mathscr{J}}}^{\mathrm{(1)}}(N) & \equiv  & {\mathscr{J}}\mathrm{(2}N,{{\lambda }}_{{\rm{m}}})-{\mathscr{J}}(N,{{\lambda }}_{{\rm{m}}})\\  & = & {\int }_{0}^{{{\lambda }}_{{\rm{m}}}}{f}^{\mathrm{(1)}}({\lambda };N)d{\lambda }\end{array}$$

19b $$\begin{array}{ccc}{{\mathscr{J}}}^{\mathrm{(2)}}(N) & \equiv  & {{\mathscr{J}}}^{\mathrm{(1)}}\mathrm{(2}N)-{{\mathscr{J}}}^{\mathrm{(1)}}(N)\\  & = & {\int }_{0}^{{{\lambda }}_{{\rm{m}}}}{f}^{\mathrm{(2)}}({\lambda };N)d{\lambda }\end{array}$$

We suppress indication of dependence on *λ*_m_ for ${{\mathscr{J}}}^{\mathrm{(1)}}$ and ${{\mathscr{J}}}^{\mathrm{(2)}}$ because these are independent of *λ*_m_ for sufficiently large *λ*_m_. The first and second integral differences are evaluated using integrals of the *f* differences, so they can be determined with greater accuracy than the integral ${\mathscr{J}}(N,{\lambda }_{{\rm{m}}})$.

We evaluate ${\mathscr{J}}(N,{\lambda }_{{\rm{m}}})$ directly (i.e., via quadrature according to equation (15)) only for *N*_0_, where we have the largest number of quadrature points. Then we determine ${\mathscr{J}}(N,{\lambda }_{{\rm{m}}})$ for larger *N* by adding the ${{\mathscr{J}}}^{\mathrm{(1)}}({N}_{0})$ and ${{\mathscr{J}}}^{\mathrm{(2)}}({N}_{j})$ differences, evaluated via numerical integration of *f* ^(1)^ and *f* ^(2)^ according to equation (19). Specifically:

20a $${\mathscr{J}}({N}_{1},{{\lambda }}_{{\rm{m}}})={\mathscr{J}}({N}_{0},{{\lambda }}_{{\rm{m}}})+{{\mathscr{J}}}^{\mathrm{(1)}}({N}_{0})$$

20b $${\mathscr{J}}({N}_{2},{{\lambda }}_{{\rm{m}}})={\mathscr{J}}({N}_{1},{{\lambda }}_{{\rm{m}}})+{{\mathscr{J}}}^{\mathrm{(1)}}({N}_{0})+{{\mathscr{J}}}^{\mathrm{(2)}}({N}_{0})$$

20c $${\mathscr{J}}({N}_{n},{{\lambda }}_{{\rm{m}}})={\mathscr{J}}({N}_{n-1},{{\lambda }}_{{\rm{m}}})+{{\mathscr{J}}}^{\mathrm{(1)}}({N}_{0})+\sum _{k\mathrm{=0}}^{n-2}{{\mathscr{J}}}^{\mathrm{(2)}}({N}_{k})$$

Thus, in Fig. 2 the only data subject to quadrature are those represented by lines joining points. The other data, represented by symbols in the plot, are used only to compute the higher-order differences.

### Simulation details

We performed canonical-ensemble molecular simulations of the Sutton-Chen potential reported in (I), which was obtained there by fitting to DFT energies of selected configurations of the bcc and hcp phases at 6000 K and 360 GPa; this model truncates interactions at a distance of 6 Å, which puts a lower bound on the system size that may be simulated for a given density. We generated configurations using MD with an Andersen thermostat. All atom velocities were randomized by sampling a Maxwell-Boltzmann distribution every 5000 steps (5 ps).

The relative coordinates of coupled atoms were handled within a multi-timestep integration framework, with the spring force handled analytically in the inner loop. For large values of *w*, the period of the harmonic spring matches the data collection interval such that atoms tend to be at the same relative position each time data is collected, leading to highly correlated results. To break this, we randomized the relative velocity between the atoms in each pair.

For very large values of *w*, the multi-timestep approach fails to properly sample the appropriate distribution of relative coordintes for the coupled pairs. As a remedy, we held r_12_, the vector between atoms 1 and 2, at a fixed value during MD integration and then sampled each component of r_12_ vector from a Gaussian distribution centered at r_12_ = 0 with width *σ* = (*k*_*B*_*τ*/2*εw*)^1/2^. Although the Gaussian distribution perfectly samples the probability due to the spring energy, the iron energy is ignored when selecting new positions. Consequently, we must consider the new positions to be trials that we would accept or reject with Metropolis Monte Carlo (MC) criteria:

21 $${P}_{{\rm{acc}}}=\,{\rm{\min }}(\mathrm{1,}\,{e}^{-\beta ({U}_{\text{Fe},\text{new}}-{U}_{\text{Fe},\text{old}})})$$

Simulations were performed for a temperature *T* = 7000 K sampling an NVT ensemble with volumes *V* selected to result in a pressure of 360 GPa for the given temperature and crystal structure (as given from short NPT runs in LAMMPS): 6.65 Å^3^/atom for the bcc phase, and 6.616 Å^3^/atom for hcp. We note that according to (I), the difference in volumes of the two phases at this temperature and pressure when simulated using the DFT potential is about 50% larger than we obtained for the Sutton-Chen potential used here.

Following the scheme outlined in the previous section, system-size doublings were performed to yield free energies from *N* = 512 to 65,536 (seven doublings) for the bcc phase, and *N* = 192 to 24,576 (seven doublings) for the hcp phase (which employs an orthorhombic unit cell with a 4-atom basis). We note that further halving of the smallest systems would result in a simulation volume that is smaller than twice the cutoff radius of the potential, leading to inaccurate results, so these are the smallest systems sizes we can use in this sequence. The shape ratio of the simulation supercell (if unit cell were considered as cubic) cycles from oblate to cubic to prolate and back to oblate in successive doublings. The supercell shapes are recorded along with the system sizes in Table 3. As indicated in the table, we refer to the system sizes from smallest to largest as *N*_0_, *N*_1_, …, *N*_7_, such that *N*_*j*_ = 2^*j*^*N*_0_.

**Table 3:** System sizes and integration parameters used in this study. In each case, simulations were performed using the number of atoms indicated as *N*_*j* + 1_ (=2*N*_*j*_).

*j*	bcc				hcp			
	*N*_*j*_ → *N*_*j* + 1_	Supercell	*λ*_m_	*n*_*λ*_	*N*_*j*_ → *N*_*j* + 1_	Supercell	*λ*_m_	*n*_*λ*_
0	512 → 1024	8 × 8 × (4 → 8)	19.0	227	192 → 384	6 × 4 × (2 → 4)	19.0	201
1	1024 → 2048	(8 → 16) × 8 × 8	19.0	130	384 → 768	(6 → 12) × 4 × 4	19.0	111
2	2048 → 4096	16 × (8 → 16) × 8	10.0	52	768 → 1 536	12 × (4 → 8) × 4	10.0	50
3	4096 → 8192	16 × 16 × (8 → 16)	10.0	43	1536 → 3072	12 × 8 × (4 → 8)	9.6	41
4	8192 → 16384	(16 → 32) × 16 × 16	10.0	42	3072 → 6144	(12 → 24) × 8 × 8	9.6	41
5	16384 → 32768	32 × (16 → 32) × 16	10.0	7	6144 → 12288	24 × (8 → 16) × 8	9.6	20
6	32768 → 65536	32 × 32 × (16 → 32)	8.4	5	12288 → 24576	24 × 16 × (8 → 16)	9.6	14

Integration is performed for *λ* from 0 to *λ*_m_, using *n*_*λ*_ steps, and yields Δ*A*(*N*_*j*_) ≡ *A*(*N*_*j* + 1_) − *A*(*N*_*j*_). For hcp, *λ* steps are equally spaced, but in bcc the separation varies along the path.

To obtain correct results, it is necessary for the atoms to explore all positions in the simulation box to the extent allowed by the constraint function *ϕ*. Diffusion is not sufficiently fast to ensure this outcome, so additional MC trials were performed in which two atoms selected at random are considered to swap positions; swaps were considered for neighboring atoms within 2 to 6 Å separation (depending on *w*). Acceptance of the trial was made according to the usual Metropolis recipe^7^. These trials require no recalculation of the iron potential energy (*U*(r^2*N*^)), and involve changes only in the interaction of each atom with its partner via *ϕ*, so they are computationally inexpensive and could be performed repeatedly; 10*N* MC swap trials were performed every 10 fs, after completing the MD/MC acceptance decision.

For each system-doubling, simulations were performed for values of *λ* from 0 to *λ*_m_, and the integrand specified by equation (12) was averaged (separate averages were kept for *U*, *ϕ*, and *dϕ*/*dw*). The specific choice of *λ*_m_, and number of quadrature points *n*_*λ*_ for each *N*-doubling process is recorded in Table 3. To keep computation time manageable, the *n*_*λ*_ was reduced about in half for each size doubling (though in some cases additional points were used to ensure the integrand was resolved sufficiently). Each simulation sampled 1 × 10^5^ MD steps, or 100 ps total; this is after 11.5 ps of equilibration. Hence, the *N*_0_ → *N*_1_ system-doubling involved 100 ps × 227 *λ* steps, totaling 22.7 ns of sampling, and the *N*_6_ → *N*_7_ case performed a total of about 500 ps of sampling.

Simulation averages for each *λ* were collected into 100 blocks for error analysis; the covariance between successive blocks was examined to ensure they were uncorrelated. Stochastic uncertainties were computed as one standard deviation of the mean (68% confidence level). Inaccuracy (systematic error) in Δ*A* arising from the trapezoid-rule numerical integrations was estimated by removing half (or more) of the quadrature points used to compute each integral. In all cases, the inaccuracy is found to be smaller than the stochastic uncertainty.

All calculations reported here were performed using the Etomica molecular simulation package^8^. Tests were conducted of configuration energies to ensure that the energy potential function was consistent with that implemented in LAMMPS, which was used by the authors of (I) in their studies of the Sutton-Chen model. Additionally, we tested the free energy-calculation framework by applying the method to the Lennard-Jones model; the result obtained this way matched accurate values that we obtained independently using more conventional methods^2^.

All data generated or analysed during this study are included in this published article.
